# Theoretical Study on Gaussian Polymer Chains for Spin–Echo Small-Angle Neutron Scattering

**DOI:** 10.1021/acs.jpca.4c07859

**Published:** 2025-02-13

**Authors:** Tengfei Cui, Xiang-qiang Chu

**Affiliations:** † Graduate School of China Academy of Engineering Physics, Beijing 100193, China; ‡ Department of Physics, City University of Hong Kong, Hong Kong SAR 999077, China; § Shenzhen Research Institute, City University of Hong Kong, Shenzhen 518057, China

## Abstract

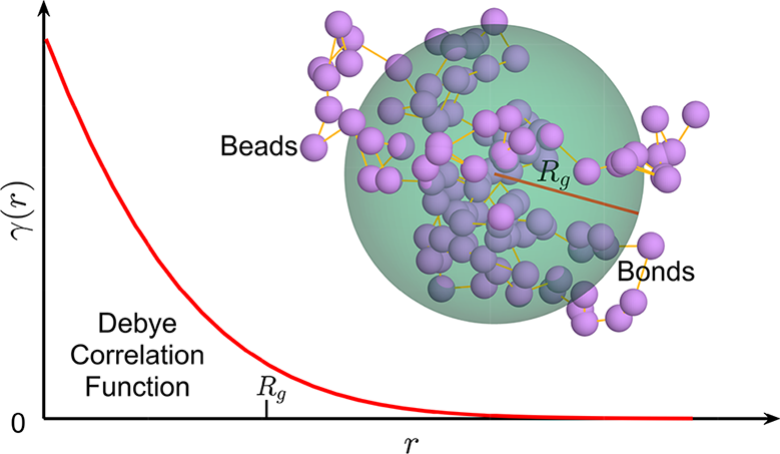

This study develops a generalized method for applying spin–echo small-angle neutron scattering (SESANS) to the structural analysis of polymers. Starting from the theoretical framework of SESANS, we developed real space correlation functions for the Gaussian chain model systems consisting of chains with many beads. Further molecular dynamics (MD) simulations affirm that the functions derived by our proposed theoretical work can accurately predict the radii of gyration of polymer chains, which bring straightforward insight of SESANS measurements. This work will enable a broader application of SESANS in soft matter analysis.

## 1 Introduction

Small-angle neutron scattering (SANS) is one of the most mature and widely used techniques in studying the microstructure of samples ranging from tens to hundreds of nanometers. Spin–echo small-angle neutron scattering (SESANS) is a technique circumvents the collimation problem in traditional SANS technique by introducing the manipulation of neutron spins through its Larmor procession in magnetic fields, which allows the expansion of spatial detection range to 20 microns.^1−3^ With the advantages of SESANS, a number of successful applications have been conducted in the field of soft matter,^4^ including the discovery of the mechanism of formation of mesoporous silica in solution,^5^ as well as the characterization of microstructures in oil–water microemulsions.^6^ Furthermore, SESANS is more suitable for studies with strong scattering or thicker samples compared to SANS, as it allows direct characterization of concentrated synthetic dispersions without the need to dilute them.^7^ Moreover, numerous reports have emerged regarding research on the microscopic mechanisms of colloidal particles self-assembling into colloidal strings in recent years.^8−11^ The complex interactions between particles drive colloidal self-assembly into a rich variety of string-like structures, whose sizes can reach several micrometers or even higher orders of magnitude. As a result, SESANS holds great potential for studying colloidal strings’ self-assembly mechanisms.

**Figure 1:**
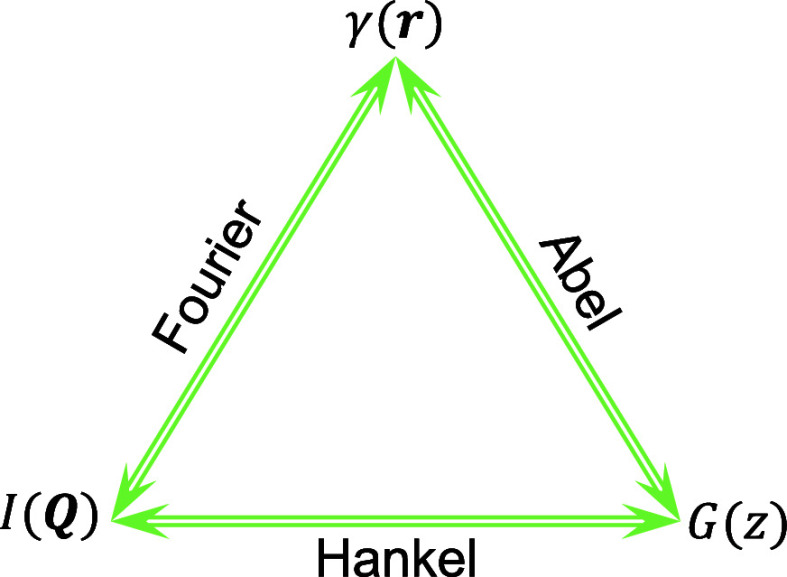
Fourier–Abel–Hankel cycle. Any two of the three functions can be transformed to each other. ***r*** represents the position of a particle in the real space. ***Q*** is the wavevector transfer in the reciprocal space. *z* means the spin–echo length in SESANS.

Currently, there is considerable interest in studying the correlation between particles in polymers or macromolecules, and the correlation function γ(***r***) of the particle scattering length density spatial distribution ρ(***r***) is usually taken as one of the decisive factors among the studies. The Debye type correlation between particles is included in the γ(***r***), which contains information about the microstructure of a sample. The famous Fourier-Abel-Hankel cycle (Figure 1) provides us with detailed information regarding how correlated factors are transformed.^12^ The γ(***r***) is the inverse Fourier transform of *I*(***Q***), which is the neutron scattering intensity in SANS. The projected correlation function *G*(*z*), which is directly related to the neutron polarization *P*(*z*) collected by SESANS method, is the Abel transform of γ(***r***). In addition, *I*(***Q***) and *G*(*z*) can be transformed to each other by the Hankel transform.

**Figure 2:**
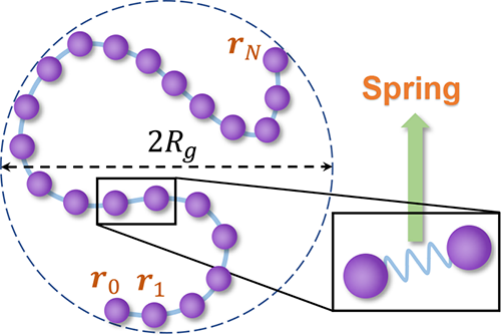
Illustration of a Gaussian chain. Beads are connected by springs. Beads and springs represent polymer segments and chemical bonds, respectively.

**Figure 3:**
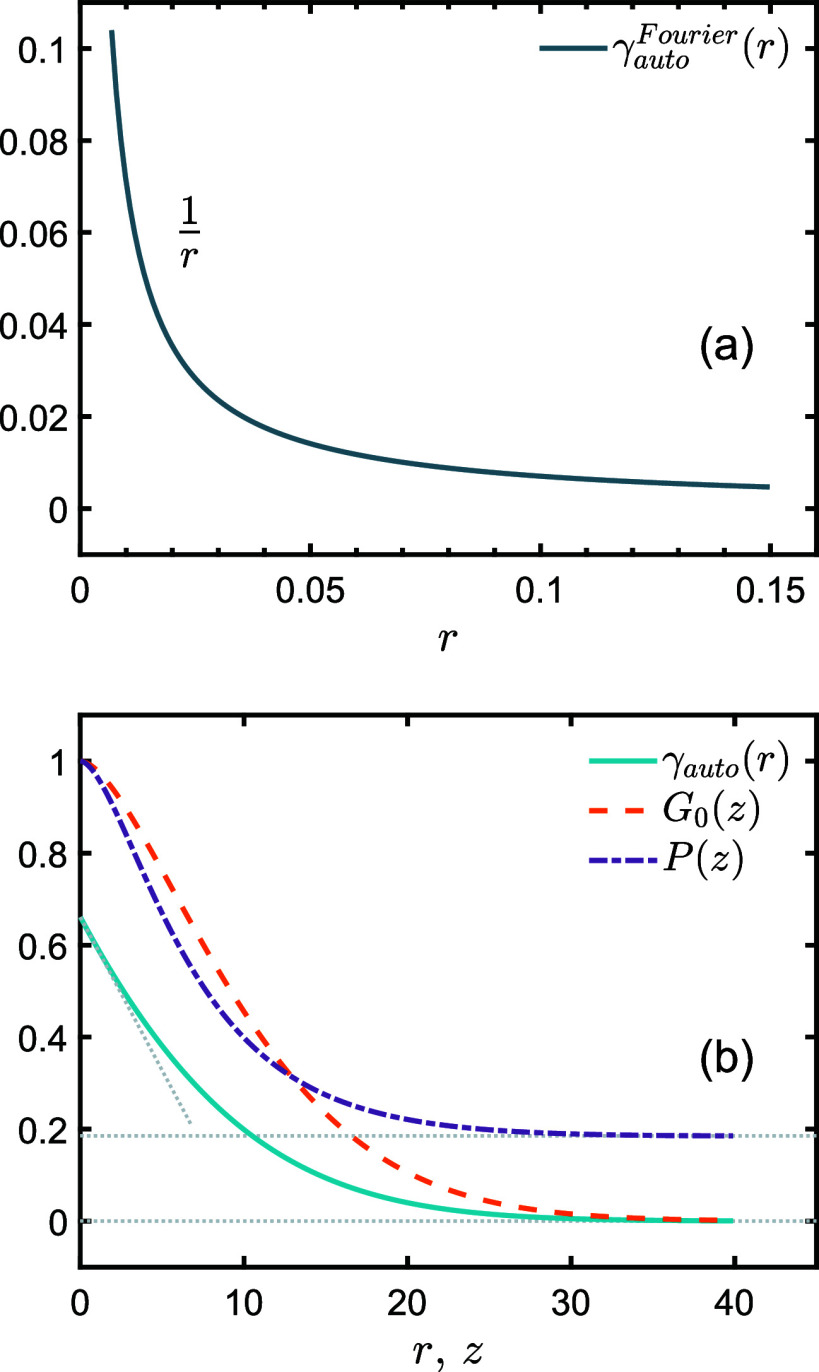
(a) Function curve of  tends to infinity as *r* tends to 0. (b) Solid line is the autocorrelation function γ_auto_(*r*), the dashed line is the normalized SESANS projected correlation function *G*_0_(*z*), the dashed-dotted line is the neutron polarization *P*(*z*). The gray dotted line is used to guide the line of sight. All curves in (a) and (b) take parameter *R*_g_ = 15 (arbitrary unit).

To understand the particle correlations in soft matter from the scattering spectrum, theoretical models have been developed to determine its microscopic information. The Gaussian chain (Figure 2) model is one of the fundamental theories for studying polymer materials. For example, the amorphous polymer chains, are well described with the Gaussian model as pliable and entangled strings in a molten state or in solution under Θ temperature, which is the specific condition at which the interaction between the polymer and the solvent is zero.^13^ On the experimental side, over the past decades, the Gaussian chain model has been widely used in analyzing the SANS data obtained on polymer materials to study their structural properties.^14−17^ It is reported that a fractal model has been applied to analyze the data measured by the SESANS technique, characterizing that the chromatin organization in the biological cell nucleus forms a bifractal structure at multiple hierarchical scales.^3^ This fractal model can explain the fractal structure of molecules when the fractal exponent is in the range of 2 < *v* ≤ 4. The Gaussian chain model has a fractal exponent of *v* = 2, which exactly compensates for the situation that the fractal model cannot cover and demonstrates the application prospects of the model in the study of the structures of biological macromolecules such as DNA and proteins. However, the application of this model in SESANS encounters some difficulties because the correlation function *G*(*z*) obtained from the Hankel transform of *I*(*Q*) diverges in the vicinity of *z* = 0. The reason for this divergence is that the Gaussian chain’s form factor, which is proportional to *I*(*Q*), decays with *Q*^–2^, similar to the divergence exhibited in the inverse Fourier transform of the form factor (Figure 3a).^18^ In most cases, a *Q*_max_ limit was used as a truncation of the Hankel transform as the maximum *Q* value in SESANS measurements is limited due to the detector size, which makes the above transformation process less accurate, as well as the truncated *G*(*z*) cannot be normalized at *z* = 0.^19^ What’s more, divergence at *z* = 0 may also be addressed by other approximate calculation methods. For instance, the formula could be modified within a small range of *z*.^20^ The specific methods of modification, however, require further investigation.

In this work, we introduce a new method for deriving correlation functions of the Gaussian chain model for SESANS measurements. The correlation functions solved by this method has been proved to be nondivergent. In addition, our molecular dynamics simulation results provide us more evidence for the feasible of this new model in SESANS by exhibiting comparasion between the fitted radius of gyration *R*_g_ and the actual *R*_g_ value. Our work offers a more in-depth understanding of the SESANS spectrum.

## 2 Methods

### 2.1 Theoretical Framework of SESANS

SESANS spectrometers are designed to measure the variation of neutron polarization *P*(*z*) by manuplating the incident and exit neutron Larmor procession.^1,2,21^ Consequently, the structural information on the measured sample is encoded in *P*(*z*), which was proved to be a function about *G*(*z*). Consider a sample with thickness of *l*, exposed to a neutron beam with cross section area *S* and a fixed neutron wavelength of λ. The illuminated volume of the sample is represented by *V* = *lS*. The relationship between *P*(*z*) and *G*(*z*) is given by^22^
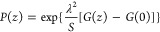
1which is an expression in the presence of multiple scattering and single scattering is its first-order expansion.^23^ The *G*(*z*), in eq 1, can be normalized as *G*_0_(*z*) = *G*(*z*)/*G*(0).

### 2.2 Gaussian Polymer Chain

A Gaussian chain is typically represented as a sequence of *N* + 1 beads linked by harmonic springs, where the beads are considered as point-like entities (Figure 2). This model posits that monomers within a chain interact without long-range forces, assuming configurations through random walks. This approximation is based on the premise that the dimensions of a molecule significantly exceed those at the atomic scale. The distribution of effective bond lengths *b* within the chain adheres to a Gaussian distribution shown as
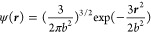
2

This leads to the conclusion that *b* is related to the squared distance between adjacent beads in the chain by the equation ⟨***r***^2^ ⟩ = *b*^2^.

Furthermore, the Debye correlation function γ(***r***) is defined as^24,25^
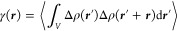
3where Δρ (*r′*) represents the difference in scattering length density (SLD) between the local position ***r****′* within the sample and the average SLD across the entire sample.^26^ ⟨·⟩ denotes the ensemble average, which is averaging over all possible conformations of a chain in the Gaussian chain model.

Moreover, in the context of SANS, the form factor for Gaussian chains^27^
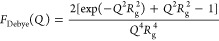
4is identified as the Debye function. The function *F*_Debye_(*Q*) is exclusively dependent on the radial direction of ***Q*** indicating that a specimen containing Gaussian chains is isotropic. In the Debye function, there exists solely a single parameter *R*_g_, which serves to characterize the size of an individual chain. Should one aim to explore more details within a single chain, it becomes imperative to consult some other, comparatively more complex form factor models.^28^ The inverse Fourier transform of *F*_Debye_(*Q*) yields^29^
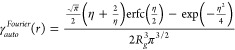
5where erfc(*x*) is the complementary error function and η = *r*/*R*_g_. The “*auto*” denotes a function that is exclusive to the conformation of a single chain, not the collective structure of multiple chains. The γ_auto_(*r*) can be regarded as a real space form factor. As we all know, a correlation function can be interpreted as a probability density of the distribution of particles in space. However, the divergence of eq 5 near *r* = 0 makes this interpretation of probability density unreasonable.

## 3 Results and Discussion

### 3.1 Theoretical Results

In this work, a model is created. The model represents a molten polymer system that contains *N*_c_ chains, with a proportion of deuterated chains denoted by ϕ. The scattering lengths for hydrogenated and deuterated monomers are denoted as β_H_ and β_D_, respectively. Generally, for a homogeneous particle, the function γ(*r*) describes the shared volume of the particle and its phantom when the phantom is shifted by a distance *r* from the initial position.^22^ However, this method does not apply to the Gaussian chain model because it neglects the volumetric presence of monomers within each chain. Therefore, we derived the Gaussian chains’ γ(*r*) function based on the initial definitions of eqs 2 and 3,
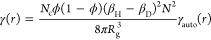
6which is proportional to the autocorrelation function γ_auto_(*r*). Since polymer chains are not fixed structures within soft matter systems, an ensemble average across all possible conformations has been performed on eq 6. The γ_auto_(*r*) is defined as a dimensionless function and its detail follows:
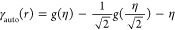
7where η = *r*/*R*_g_ for *r* ≥ 0,
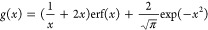
8and erf(*x*) is the error function (*g*(*x*) is defined to simplify eq 7). Our results make improvements on eq 5.

What’s more, *G*(*z*) can be obtained by performing the Abel transform on eq 6. And *G*(0) = *N*_c_ ϕ (1 – ϕ)(β_H_ – β_D_)^2^*N*^2^ ln (2)/(8π*R*_g_^2^) is obtained by the Abel transform of γ(0). Therefore, the normalized function *G*(*z*) should have the following form,
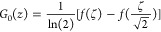
9where ζ = *z*/*R*_g_ for *z* ≥ 0,
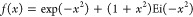
10and Ei(*x*) is the exponential integral function (*f*(*x*) is defined to simplify eq 9).

Moreover, the polarization eq 1 can be rewritten as
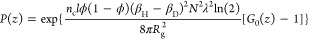
11where *n*_c_ = *N*_c_/*V* is the number density of molecular chains. Figure 3b shows the curves of γ_auto_(*r*), *G*_0_(*z*), and *P*(*z*). These curves verify their nondivergent behavior in the limit of small space. At this juncture, we have obtained an expression that can be utilized for the direct fitting of SESANS spectrum, as demonstrated in eq 11.

### 3.2 Molecular Dynamics Simulations

Additionally, in order to verify the feasibility of the results, we also performed molecular dynamics (MD) simulations to simulate the kinetic behavior of the polymer chains in the real SESANS experimental environment. We used a coarse-grained model in which every polymer chain consists of *N* + 1 interconnected beads to simulate dilute solution polymer chains at Θ temperature.^30−32^ There is an assumption in this model that the Gaussian chain beads are involved in interactions that are essentially short-ranged in nature. To capture this, an attractive Lennard-Jones (LJ) potential is applied as described by the equation:
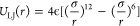
12where *r* represents the distance between the beads. The LJ potential acts between the beads, allowing them to interact with each other. It contains two parameters. ϵ characterizes the intensity of maximum attractive interaction between beads, and σ represents their distance when the interaction is zero. Furthermore, adjacent beads are linked by a finitely extensible nonlinear elastic (FENE) potential as the bond potential given by^33^
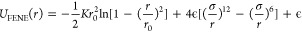
13

The first term is attractive. It is valid up to the threshold where *U*_FENE_(*r* > *r*_0_) = *∞*, thus denoting *r*_0_ as the maximum stretchability between bonded beads. The constant *K* controls the stiffness of bonds. The second term is the LJ potential, truncated at 2^1/6^ σ and shifted by the third term ϵ. The second and third terms make a repulsive potential. As a result, the three terms are combined to form an anharmonic spring potential *U*_FENE_(*r*).

The reduced units with unit length σ = 1, unit energy ϵ = 1, the Boltzmann constant *k*_B_ = 1, the mass of a bead *m* = 1 as fundamental quantities were defined in the simulations. The parameters for bonded interactions are the standard choice *K* = 30 and *r*_0_ = 1.5. The truncation distance for the LJ interactions, denoted by eq 12, is established at 2.5 and shifted to zero at the cutoff. Considering the model outlined by eqs 12 and 13, the characteristic Θ temperature is determined to be *T*_Θ_ = 3.18.^34^ Given that the conformational behavior of polymer chains in dilute solutions under Θ temperature conditions corresponds well with the Gaussian chain model, the simulation temperature was accordingly chosen as *T* = *T*_Θ_.

The simulations were performed in the MD application LAMMPS.^35^ The temperature is controlled by the Langevin thermostat to simulate the equilibrium state of the polymer chains in solution. The equation of motion for bead *i* is provided by
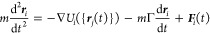
14where *U*_*i*_({***r***_*j*_(*t*)}) represents the total potential acting on the *i*-th bead due to its interactions with other beads located at {***r***_*j*_(*t*)} and at time *t*. The term Γ denotes the friction coefficient, which couples the beads to the heat bath, and ***F***_*i*_(*t*) is a white-noise random force. Due to the implicit solvent model used, random forces can be used to simulate the collision of beads with solvent molecules. It was chosen Γ = 0.5 following previous work.^36,37^ The integration time step used to numerically solve the equations of motion is Δ*t* = 0.006 with the τ = (mσ^2^/ϵ)^1/2^ = 1 being the time unit.

Four groups were simulated, and each single chain in each group contained different numbers of beads: *N* + 1 = 100, 125, 150, 175, respectively. In order to perform the simulation, there are a total of three steps in the process, which are the same for all chain lengths. Initially, a chain is placed in a square box with side lengths of 80, and allowed to equilibrate over a duration of 1 × 10^8^ Δ*t*. Subsequently, the resultant configuration from the preceding phase was replicated 50 times, introduced into a square box with side lengths of 300, and subjected to equilibration for 1 × 10^9^ time steps. Periodic boundary conditions were applied to all boxes throughout this phase. Lastly, the system was further evolved for 1 × 10^8^ steps, with trajectory data being recorded every 10,000 steps (typically exceeding 500 steps) to ensure statistical independence of configurations. Equilibrium trajectories of all particles were saved encompassing 10,000 frames for each group. Upon completion of the simulation, all parameters requiring analysis can be obtained by resolving the particle trajectories.

### 3.3 Data Analysis

The size of a polymer chain, quantified by its radius of gyration, is an important parameter in the Gaussian chain model. The radius of gyration is defined as , where ***r***_G_ represents the center of mass of a chain. In fact, the characterization of a sample’s *R*_g_ is equally important in the SESANS data analysis. We employed four methods to calculate the *R*_g_ and verify the theoretical formula by comparing the values obtained from each method. First of all, the *R*_g_ were calculated for all chains in each frame and then averaged to determine the actual radius of gyration values. In addition, the *R*_g_ of a Gaussian chain system can be theoretically estimated using the equation ,^27^ where the effective bond length *b* is defined in detail in eq 2. To determine *b* for the four groups, the squared distance between neighboring beads within each chain was calculated in a manner analogous to the determination of the actual *R*_g_. This computation involved averaging over all chains and frames extracted from the trajectories. Furthermore, we fitted the γ(*r*) data generated from the output of the LiquidLib package^38^ by using the trajectories as input and applying eq 6, with *R*_g_ as the fitting parameter. This process is analogous to fitting a real set of SESANS data using the corresponding formulas. It is important to note that there is no need to fit the *G*(*z*) data additionally, as the invertibility of the Abel transform ensures that the information from γ(*r*) and *G*(*z*) is mathematically equivalent. Finally, for any group of simulations, we used the equation  where *r*_*ij*_ is the distance between beads *i* and *j* from the MD simulation trajectory to calculate every single-chain form factor and averaged them over all chains and frames.^39^ (This calculation is completed by a Python program, and the code can be obtained from the GitHub platform.^40^) Similar to the procedural sequence of the third approach, we employ *F*_Debye_(*Q*) to conduct a parametric fitting of the form factor data, with *R*_g_ serving as the parameter. This method provides a theoretical underpinning for the reliability of the MD simulations from the vantage point of classical reciprocal space theory.

**Figure 4:**
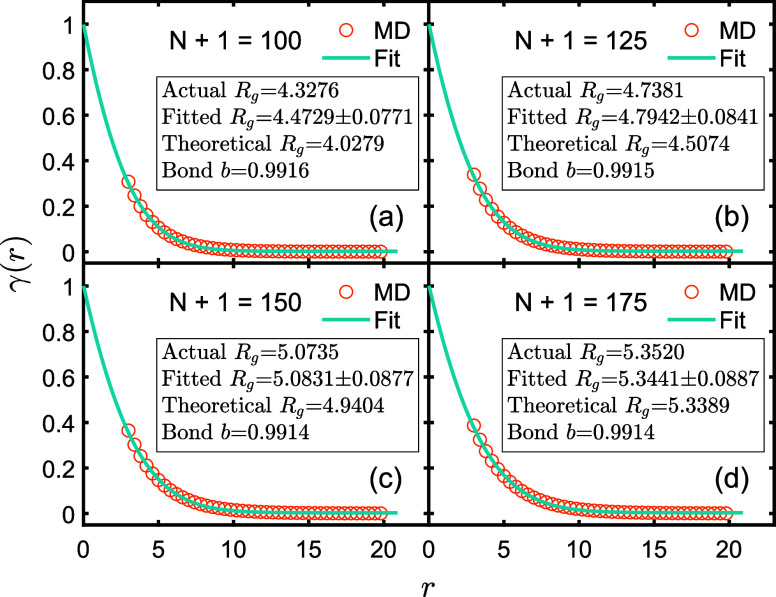
Circles represent MD simulation data of γ(*r*). Solid lines represent fitted curves by using eq 6. All data are normalized at zero.

**Figure 5:**
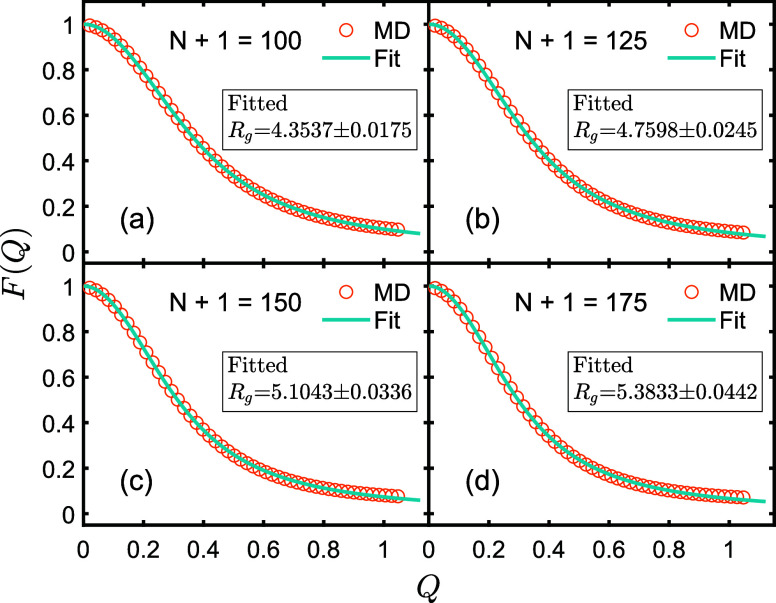
Circles represent MD simulation data of form factor *F*(*Q*). Solid lines represent fitted curves by using eq 4. All data are normalized at zero.

The outcomes from the four calculation methods for *R*_g_ as discussed above are presented in Figures 4 and 5. The actual values of *R*_g_ closely align with the theoretical values, indicating that all four sets of simulated polymer chains demonstrate Gaussian chain behavior, which is also corroborated by the fitting results in Figure 5. All the four groups of fitted values of *R*_g_ in Figure 4 deviate from the actual value by less than 3.5%, providing substantial justification for the reliability of the theory.

## 4 Conclusions

This study investigates the foundation for applying SESANS technology in the field of polymers. Beginning with the theoretical framework of SESANS, this study culminates in the derivation of the real-space correlation function γ(*r*) through a theoretical analysis of the Gaussian chain model. Furthermore, we derive analytical formulas for the projection correlation function *G*(*z*) and the neutron polarization *P*(*z*) for SESANS, both of which are functions in real space and can be intuitively understood without resorting to the concept of reciprocal space. Moreover, when analyzing SESANS data by applying the Gaussian chain model, comparing our proposed scheme with the traditional one mentioned above, our results are able to simplify the SESANS data fitting process without sacrificing accuracy, which is corroborated by the results of MD simulations, while offering a new perspective on understanding Gaussian chains. Our results will help researchers in understanding SESANS data, thereby facilitating the application of SESANS technology in polymer research.

## References

[ref1] Bouwman W. G.; Oossanen M. v.; Uca O.; Kraan W. H.; Rekveldt M. T. Development of spin-echo small-angle neutron scattering. J. Appl. Crystallogr. 2000, 33, 767–770. 10.1107/S0021889800099829.

[ref2] Kadletz E.; Bouwman W. G.; Pappas C. Radial spin echo small-angle neutron scattering method: concept and performance. J. Appl. Crystallogr. 2022, 55, 1072–1084. 10.1107/S1600576722007245. 36249505 PMC9533745

[ref3] Iashina E. G.; Bouwman W. G.; Duif C. P.; Dalgliesh R.; Varfolomeeva E. Y.; Pantina R. A.; Kovalev R. A.; Fedorova N. D.; Grigoriev S. V. Time-of-flight spin-echo small-angle neutron scattering applied to biological cell nuclei. J. Appl. Crystallogr. 2023, 56, 1512–1521. 10.1107/S1600576723007549.

[ref4] Wang T. Structural Insights into Soft Matter Materials via Spin Echo Small Angle Neutron Scattering and Small Angle Neutron Scattering. Nuclear Analysis 2024, 3, 100128 10.1016/j.nucana.2024.100128.

[ref5] Schmitt J.; Zeeuw J. J.; Plomp J.; Bouwman W. G.; Washington A. L.; Dalgliesh R. M.; Duif C. P.; Thijs M. A.; Li F.; Pynn R.; et al. Mesoporous silica formation mechanisms probed using combined spin–echo modulated small-angle neutron scattering (SEMSANS) and small-angle neutron scattering (SANS). ACS Appl. Mater. Interfaces 2020, 12, 28461–28473. 10.1021/acsami.0c03287. 32330001

[ref6] Mulder M.; Li X. X.; Nazim M. M.; Dalgliesh R. M.; Tian B.; Buijse M.; van Wunnik J.; Bouwman W. G. Systematically quantifying oil–water microemulsion structures using (spin-echo) small angle neutron scattering. Colloids Surf., A 2019, 575, 166–175. 10.1016/j.colsurfa.2019.04.045.

[ref7] Smith G. N.; Cunningham V. J.; Canning S. L.; Derry M. J.; Cooper J.; Washington A.; Armes S. P. Spin-echo small-angle neutron scattering (SESANS) studies of diblock copolymer nanoparticles. Soft Matter 2019, 15, 17–21. 10.1039/C8SM01425F. 30520930

[ref8] Townsend J.; Burtovyy R.; Galabura Y.; Luzinov I. Flexible chains of ferromagnetic nanoparticles. ACS Nano 2014, 8, 6970–6978. 10.1021/nn501787v. 24950006

[ref9] Su G.; Guo Q.; Palmer R. Colloidal lines and strings. Langmuir 2003, 19, 9669–9671. 10.1021/la035149d.

[ref10] Chao X.; Skipper K.; Royall C. P.; Henkes S.; Liverpool T. B. Traveling Strings of Active Dipolar Colloids. Phys. Rev. Lett. 2025, 134, 018302 10.1103/PhysRevLett.134.018302. 39913728

[ref11] Guo Yong B. G.; Kegel W. K. Self-assembly of isotropic colloids into colloidal strings Bernal spiral-like and tubular clusters. Chem. Commun. 2020, 56, 6309–6312. 10.1039/D0CC00948B. 32390022

[ref12] Andersson R.; Van Heijkamp L. F.; De Schepper I. M.; Bouwman W. G. Analysis of spin-echo small-angle neutron scattering measurements. J. Appl. Crystallogr. 2008, 41, 868–885. 10.1107/S0021889808026770.

[ref13] Rubinstein M.; Colby R. H. Polymer Physics; Oxford University Press, 2003.

[ref14] Debye P. Molecular-weight determination by light scattering. J. Phys. Chem. 1947, 51, 18–32. 10.1021/j150451a002. 20286386

[ref15] Arrighi V.; Gagliardi S.; Dagger A.; Semlyen J.; Higgins J.; Shenton M. Conformation of cyclics and linear chain polymers in bulk by SANS. Macromolecules 2004, 37, 8057–8065. 10.1021/ma049565w.

[ref16] Iwamoto T.; Doi Y.; Kinoshita K.; Ohta Y.; Takano A.; Takahashi Y.; Nagao M.; Matsushita Y. Conformations of ring polystyrenes in bulk studied by SANS. Macromolecules 2018, 51, 1539–1548. 10.1021/acs.macromol.7b02358.

[ref17] Robbes A.-S.; Cousin F.; Meneau F.; Jestin J. Melt chain conformation in nanoparticles/polymer nanocomposites elucidated by the SANS extrapolation method: evidence of the filler contribution. Macromolecules 2018, 51, 2216–2226. 10.1021/acs.macromol.7b02318.

[ref18] De Gennes P.-G. Scaling Concepts in Polymer Physics; Cornell University Press, 1979.

[ref19] Kohlbrecher J.; Studer A. Transformation cycle between the spherically symmetric correlation function, projected correlation function and differential cross section as implemented in SASfit. J. Appl. Crystallogr. 2017, 50, 1395–1403. 10.1107/S1600576717011979.

[ref20] Schneider R.; Belkoura L.; Schelten J.; Woermann D.; Chu B. Determination of the critical exponent η by neutron and light scattering from a binary liquid mixture. Phys. Rev. B 1980, 22, 5507–5516. 10.1103/PhysRevB.22.5507.

[ref21] Wang T.; Tu X.; Wang Y.; Li X.; Gong J.; Sun G. Design and simulations of spin-echo small angle neutron scattering spectrometer at CMRR. Nuclear Instruments and Methods in Physics Research Section A: Accelerators, Spectrometers, Detectors and Associated Equipment 2022, 1024, 166041 10.1016/j.nima.2021.166041.

[ref22] Krouglov T.; De Schepper I. M.; Bouwman W. G.; Rekveldt M. T. Real-space interpretation of spin-echo small-angle neutron scattering. Journal of applied crystallography 2003, 36, 117–124. 10.1107/S0021889802020368.

[ref23] Rekveldt M. T.; Bouwman W. G.; Kraan W. H.; Uca O.; Grigoriev S.; Habicht K.; Keller T. Elastic neutron scattering measurements using Larmor precession of polarized neutrons. Neutron Spin Echo Spectroscopy: Basics, Trends and Applications 2002, 601, 87–99. 10.1007/3-540-45823-9_9.

[ref24] Krouglov T.; Bouwman W. G.; Plomp J.; Rekveldt M. T.; Vroege G. J.; Petukhov A. V.; Thies-Weesie D. M. Structural transitions of hard-sphere colloids studied by spin-echo small-angle neutron scattering. J. Appl. Crystallogr. 2003, 36, 1417–1423. 10.1107/S0021889803021216.

[ref25] Kruglov T. Correlation function of the excluded volume. Journal of applied crystallography 2005, 38, 716–720. 10.1107/S0021889805017000.

[ref26] Li X.; Shew C.-Y.; Liu Y.; Pynn R.; Liu E.; Herwig K. W.; Smith G. S.; Robertson J. L.; Chen W.-R. Theoretical studies on the structure of interacting colloidal suspensions by spin-echo small angle neutron scattering. J. Chem. Phys. 2010, 132, 174509 10.1063/1.3422527. 20459176

[ref27] Doi M.; Edwards S. F. The Theory of Polymer Dynamics; Oxford University Press, 1988; Vol 73.

[ref28] Lages S.; Goerigk G.; Huber K. SAXS and ASAXS on dilute sodium polyacrylate chains decorated with lead ions. Macromolecules 2013, 46, 3570–3580. 10.1021/ma400427d.

[ref29] Teraoka I. Polymer Solutions: An Introduction to Physical Properties; John Wiley & Sons Inc: New York, USA, 2002.

[ref30] Grest G. S.; Murat M. Structure of grafted polymeric brushes in solvents of varying quality: a molecular dynamics study. Macromolecules 1993, 26, 3108–3117. 10.1021/ma00064a019.

[ref31] Theodorakis P. E.; Fytas N. G. Microphase separation in linear multiblock copolymers under poor solvent conditions. Soft Matter 2011, 7, 1038–1044. 10.1039/C0SM00969E.

[ref32] Theodorakis P. E.; Hsu H.-P.; Paul W.; Binder K. Computer simulation of bottle-brush polymers with flexible backbone: Good solvent versus theta solvent conditions. J. Chem. Phys. 2011, 135, 164903 10.1063/1.3656072. 22047265

[ref33] Kremer K.; Grest G. S. Dynamics of entangled linear polymer melts: A molecular-dynamics simulation. J. Chem. Phys. 1990, 92, 5057–5086. 10.1063/1.458541.

[ref34] Graessley W. W.; Hayward R. C.; Grest G. S. Excluded-volume effects in polymer solutions. 2. Comparison of experimental results with numerical simulation data. Macromolecules 1999, 32, 3510–3517. 10.1021/ma981915p.

[ref35] Thompson A. P.; Aktulga H. M.; Berger R.; Bolintineanu D. S.; Brown W. M.; Crozier P. S.; In’t Veld P. J.; Kohlmeyer A.; Moore S. G.; Nguyen T. D.; et al. LAMMPS-a flexible simulation tool for particle-based materials modeling at the atomic, meso, and continuum scales. Comput. Phys. Commun. 2022, 271, 108171 10.1016/j.cpc.2021.108171.

[ref36] Theodorakis P.; Paul W.; Binder K. Microphase separation in bottlebrush polymers under poor-solvent conditions. Europhys. Lett. 2009, 88, 63002 10.1209/0295-5075/88/63002.

[ref37] Theodorakis P.; Paul W.; Binder K. Pearl-necklace structures of molecular brushes with rigid backbone under poor solvent conditions: A simulation study. J. Chem. Phys. 2010, 133, 104901 10.1063/1.3477981. 20849186

[ref38] Walter N. P.; Jaiswal A.; Cai Z.; Zhang Y. LiquidLib: A comprehensive toolbox for analyzing classical and ab initio molecular dynamics simulations of liquids and liquid-like matter with applications to neutron scattering experiments. Comput. Phys. Commun. 2018, 228, 209–218. 10.1016/j.cpc.2018.03.005.

[ref39] Als-Nielsen J.; McMorrow D. Elements of Modern X-ray Physics, 2nd ed.; Wiley, 2011.

[ref40] Niu G. Structure-Factor-Form-factor. Retrieved from [https://github.com/little-bull/Structure-Factor-Form-factor.git], 2024; Code for calculating structure factor and form factor.

